# On the Acrodynia, or Epidemic of Paris

**Published:** 1831-01-01

**Authors:** 


### XXXIII.

### On the Acrodynia, or Epidemic which has reigned in Paris and its Environs, since the Year 1828. By M. Chardon, Jun.

This epidemic, although it has not consigned so many of our continental neighbours to their long homes as the political epidemy of July, 1830, has yet been productive of no trifling misery both in Paris and its neighbourhood, for two years past, nor are its ravages yet at an end. It is one of those mysterious visitations which evidently originate in exhalations from the earth, though the nature of these effluvia is entirely unknown.

This malady, generally unaccompanied by pyrexia, affects in a peculiar manner, the nervous system—especially by a painful sense of formication in the hands and feet—as also a numbness that invades first the members, and spreads afterwards to the trunk itself. The cellular tissue of the cutaneous structures becomes affected—the hands and feet swell—and œdema invades the face and several other parts of the body. The formication and painful numbness of the extremities are so characteristic of the complaint—that, both in Paris and in the country, it is known by the name "*mal des pieds et des mains.*" On this account our author has given it a more classical title — ACRODYNIA — or pains in the hands and feet. It appears that this mysterious epidemic has affected immense numbers in France, and was not very dissimilar to the Dandy fever of the West Indies, which spread over so many islands of late years.

The complaint usually commenced by a sense of the most painful formication in the fingers and ankles, spreading thence to the arms, thighs, and even the trunk. The sensation was compared to a thousand punctures made with the point of a lancet. An intense heat aggravates the sufferings of the patients, and obliges them to keep their feet out of bed. The perversions of sensibility are extremely various and distressing. Many cannot put their feet to the ground without feeling as if they were treading on the points of pins or needles, &c. The muscular powers of the members are also much affected. Many people could scarcely move their lower extremities without the greatest suffering. The fingers were usually in a state of permanent contraction. Subsultus tendinum was no unusual phenomenon, together with cramps, spasms, and other torments.

An affection of the mucous membranes was not an accidental accompaniment, but a characteristic feature of the epidemic. Sometimes it amounted to acute gastro-enteritis, was attended with smart fever, and was only of short duration. The functions of the digestive organs were always much disordered. Cholera morbus was occasionally developed in the course of the disease. Inflammation of the conjunctiva was no unusual concomitant, as was also pulmonary catarrh. In short all the mucous membranes were more or less affected. Dysury and gonorrhœa were not unusual. The skin was affected in a great variety of ways—but an intolerable sense of stinging, succeeded by erythema, were the usual precursors of the different complaints. Eruptions of all kinds took place—some resembling urticaria, some like small pox, and others like chicken pox, pemphygus, &c. In fact there was no end to the cutaneous affections.

The next train of phenomena consisted in the establishment of dropsical effusions in various parts of the body—œdematous, ascitic, and anasarcous. Abundant perspirations were often seen to occur in a periodical manner. Sleep could not be obtained on account of the irritation and pains. The senses were often suddenly and strangely affected. Some lost sight, or hearing, or smelling almost instantaneously. The duration of the disease was as various as its symptoms. Some patients recovered in a few weeks — others required several months for convalescence. There are many who suffer to this day from attacks in the early part of 1828. The prognosis was favourable when the disorder of the internal organs was slight —unfavourable in opposite conditions. Immense numbers lost their lives by the epidemic or by the consequences which it left behind—especially dropsy.

In several of the hospitals, the most rigorous dissections were made, but no light was thrown upon the disease by the scalpel of the anatomist. M. Louis, at La Charité, examined very carefully some who fell victims to the most exquisite forms of the epidemic, and could not find any thing to account for the disease itself or the death of the patients. In some of the public establishments, however, it is stated that portions of the spinal marrow were found softened and partially disorganized.

*Treatment.* As may be imagined, the means of curing or alleviating this strange disease were numerously employed. Venesection was sometimes found useful, especially in the beginning, and where symptoms of congestion about the head existed. Under all other circumstances the relief was only momentary—or none at all. Leeches to the abdomen produced no mitigation of the colic and diarrhœa, though the doctrines of Broussais would assure us of their efficacy. They were much more useful when applied along the spinal column. So were cupping-glasses with scarification. Warm bathing was beneficial —especially vapour and sulphur baths. The distressing sense of formication was occasionally soothed by saturnine lotions and even by unctuous applications. Moxas applied to the spine were advantageous in a few instances. But the most remarkable benefit was obtained by blisters, especially when they were made to produce a purulent discharge. They were applied to the most painful parts, or to the track of the spine. They were dressed with the antimonial ointment.

Emetics were administered internally at the beginning, with some advantage. M. Cayol employed purgatives combined with opiates, and it was said, with success. It is needless to detail the catalogue of internal medicines which were administered by different practitioners and at different hospitals. Few, if any of them, did good, as the disease generally ran its course in spite of physic.

*Causes.* Although this epidemic spared no class, yet it chiefly affected the poor. Among the troops, the officers suffered little, compared with the men. Males were much more numerously affected than females. The bread, the wine, and other species of provisions, were alternately accused as causes of the epidemic, but without the least foundation. The state of the air was suspected, with more justice; but no appreciable vitiation of the atmosphere was present, except a peculiar bad smell which infested several places where the epidemic prevailed, both in the neighbourhood of Paris and in some other places. But the true nature of the cause of this epidemic, as well as that of many others, remains in impenetrable obscurity.

In respect to the principal or primary seat of the disease itself, there has been much diversity of opinion. It was, at first, considered as rheumatismal—again, the spinal marrow was looked on as the principal seat of the malady, as evinced by the formication, the paralysis, and various other lesions of the nervous system. This opinion was strengthened by the fact, or at least the belief, that those remedies which were applied to the spine had most influence on the disease, such as leeches, blisters, frictions, &c. Strychnine considerably aggravated the symptoms. But the fact is, that the skin, the cellular tissues, the mucous membranes, the lymphatics, and various other structures of the body, were affected in this epidemic, and therefore it is not possible to confine its seat to any one organ or part.

Many facts are brought forward to shew that the disease was communicable, or in other words, contagious. This we have no reason to doubt, since there are few epidemics that do not evince this character at some period of their course. The French physicians, however, have not subjected themselves to criticism, as many English have done under similar circumstances, by broaching the doctrine that the epidemic was *imported* from abroad!

It is hardly necessary to say that almost every kind of treatment was equally unsatisfactory—we might say, with no great violence to truth—INEFFECTUAL!

---

XXXIV.

